# Widespread chytrid infection across frogs in the Peruvian Amazon suggests critical role for low elevation in pathogen spread and persistence

**DOI:** 10.1371/journal.pone.0222718

**Published:** 2019-10-16

**Authors:** Imani D. Russell, Joanna G. Larson, Rudolf von May, Iris A. Holmes, Timothy Y. James, Alison R. Davis Rabosky

**Affiliations:** Department of Ecology and Evolutionary Biology and Museum of Zoology (UMMZ), University of Michigan, Ann Arbor, Michigan, United States of America; Universitat Trier, GERMANY

## Abstract

Outbreaks of emerging infectious diseases are becoming more frequent as climate changes wildlife communities at unprecedented rates, driving population declines and raising concerns for species conservation. One critical disease is the global pandemic of chytridiomycosis in frogs, which can be caused by the fungal pathogen *Batrachochytrium dendrobatidis* (Bd). Although there is clear evidence for Bd-induced mortality across high-elevation frog communities, little attention is given to the role of lowlands in Bd’s persistence and spread because low elevations are assumed to be too warm to harbor significant levels of Bd. Here, we report widespread Bd infection across 80 frog species from three sites in the lowland Peruvian Amazon, an area with no documented Bd-related amphibian declines. Despite observing no clinical signs of infection in the field, we found that 24–46% of individuals were infected per site (up to ≈105,000 zoospore equivalents per frog) by three Bd strains from the global pandemic lineage (Bd-GPL). We also found collection site and seasonal effects to be only weak predictors of Bd prevalence and load, with lower elevation and drier habitats marginally decreasing both prevalence and load. We found no further effect of host phylogeny, ecotype, or body size. Our results showing high and widespread prevalence across a lowland tropical ecosystem contradict the expectations based on the global pattern of pathogenicity of Bd that is largely restricted to higher elevations and colder temperatures. These findings imply that the lowlands may play a critical role in the spread and persistence of Bd over time and space.

## Introduction

The ecological and evolutionary dynamics of emerging infectious wildlife diseases (EIWDs) are gaining more attention due to their significant impact on biodiversity and ecosystem services. Mycoses (diseases caused by fungi) are increasing in frequency at a rate greater than diseases of other pathogen groups [1]. Some of the most destructive wildlife mycoses currently on the rise are caused by “cold-loving” fungi, whose optimal thermal range lies within cooler temperatures. As weather events become more extreme, the stress caused by local temperature changes can increase a population’s susceptibility to disease and mortality [2]. Significant cold-loving mycoses include white-nose syndrome, which has caused massive bat population declines in the eastern U.S. [3], and chytridiomycosis, which has caused frog population declines worldwide [4]. Chytridiomycosis, which is caused by the chytrid fungus *Batrachochytrium dendrobatidis* (Bd) [5], is a classic example of a cold-tolerant pathogen, causing severe frog population declines in moderate to high-elevation areas [6–8], deemed disease “hotspots”.

The community-wide dynamics of chytrid infection can be complicated, as some populations are driven to extirpation while others may show no signs of disease. However, these “carrier” populations can be critical reservoirs for future outbreaks [9, 10]. Although the specific mechanism that triggers Bd epizootics has not yet been identified, the main hypothesized drivers of these population-level differences cluster into three classes: climate, host susceptibility, and pathogen virulence. Climate (specifically, temperature and elevation) is often regarded as the main determinant of where Bd can survive and reproduce. Bd epizootics appear to be restricted to higher elevations and cooler temperatures, and have been documented in the montane regions of Panama, Costa Rica, and Australia [6], as well as the Sierra Nevada [7], Pyrenean [11], and Andean mountain ranges [8]. In addition to the effect of climate on disease dynamics, different host populations and species may have varying levels of susceptibility to Bd, potentially conferred by the interaction between genetics [12, 13], microbiome [14], and ecotype [15]. Lastly, pathogen virulence can determine Bd prevalence and intensity in a community and has been shown to increase when Bd is within its optimal thermal range (17–25°C [16]) and decrease as temperatures exceed this [4]. Bd virulence also has the potential to differ by lineage and strain [17], with the global pandemic lineage (Bd-GPL) tending to exhibit higher virulence than endemic lineages [18], but strain-to-strain differences in virulence within the global lineage is variable depending on the isolate [19].

Here, we identify an unexpectedly high level of Bd prevalence across multiple clades of frogs from the lowland Peruvian Amazon, an area with no documented frog declines that has been predicted to be disease-free due to its warm temperatures [16, 20–22]. This study is pivotal because previous Bd studies in greater Amazonia have concentrated on Bd hotspots in the Eastern Andes [8], and those focused on lowland Peru have found very low infection prevalence [23, 24]. Those findings have been used to inform ecological niche models [20, 22, 25], which then predict very little to no Bd presence in warm, lowland areas despite their proximity to higher-elevation disease hotspots. Despite extreme frog species diversity, Bd reports are scarce over the western Amazonian lowlands, and infection data are imperative to better inform future niche models and predictions. Identifying the key factors that allow Bd to exist in lowland Amazonian forests will help us compare disease dynamics in high versus low prevalence sites and aid in the implementation of future disease mitigation strategies.

**Fig 1:**
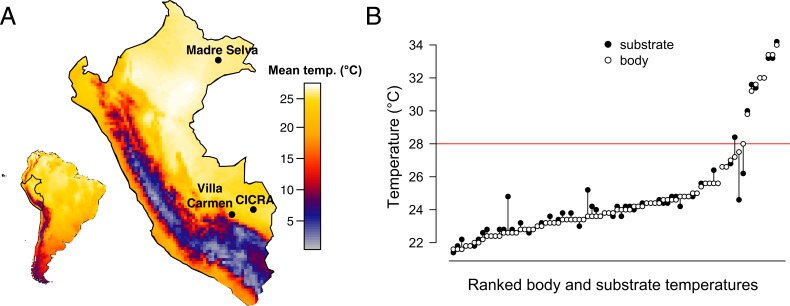
Climate and microhabitat profiles show higher temperatures than optimal for chytrid fungus. (A) Heat map of Peru (inset South America) displaying mean annual temperature data from worldclim.org (accessed January 2018) shows that all three collection sites (labeled black points) experience warm temperatures above the optimal temperature for Bd (17–25°C). (B) Paired frog body and substrate temperatures (*N* = 78) from November—December 2017 at Los Amigos show tight matching of body and microhabitat temperatures, which can be much higher than the CTmax of chytrid fungus (horizontal red line).

**Fig 2:**
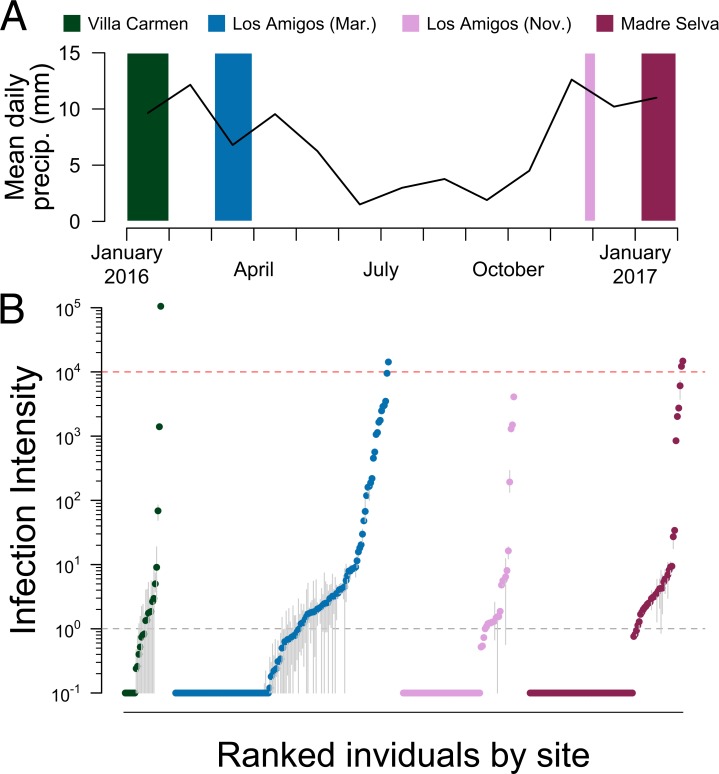
Significant infection loads were present at all time points and sites during the wet season. (A) Mean daily precipitation (mm) at Los Amigos (black line) averaged by month from January 1, 2016—February 1, 2017. Madre Selva and Villa Carmen have some differences in wet season length and intensity, but January is deep into the wet season at all localities. Colored blocks are placed and size scaled to the number of days spent on each collection expedition, and colors correspond to sites shown in panel B. (B) Log_10_-transformed infection load (note log scale on the y-axis), calculated as each individual’s triplicate rt-PCR quantified zoospore equivalent (ZE; *N* = 324 individuals) and ranked within collection site from lowest to highest infection intensities shows that chytrid (Bd) infection is present at all collection sites and sampling times. Horizontal grey dotted line at 10^0^ denotes the load threshold for categorization as a positive infection, horizontal red dotted line at 10^4^ denotes the load threshold for categorization as a heavy infection (ZE > 10,000), grey vertical lines denote quantitative range of replicates for each individual, and points denote the individual’s mean infection load.

To further understand infection dynamics in this previously unrecognized area of Bd prevalence, we tested host and ecological traits as predictors of infection and compared these relationships over space and time. We first determined 1) the prevalence of Bd in three sites across lowland Peru, whose mean annual temperatures fall within Bd’s critical thermal range but maximum temperatures often exceed this (Fig 1), and 2) the infection load of 324 individual frogs from 80 species sampled in these sites. We then tested the effects of host and habitat traits on infection load. Lastly, we genotyped Bd samples to determine which lineages are present in lowland Peru, to better understand both virulence and geographic origin. These results provide critical information about what drives chytridiomycosis in understudied lowland systems and serve as a critical, high-infection data point in what was previously thought to be a “cold-spot” of this disease, further increasing the diversity of systems represented in the Bd literature.

## Results

**Fig 3:**
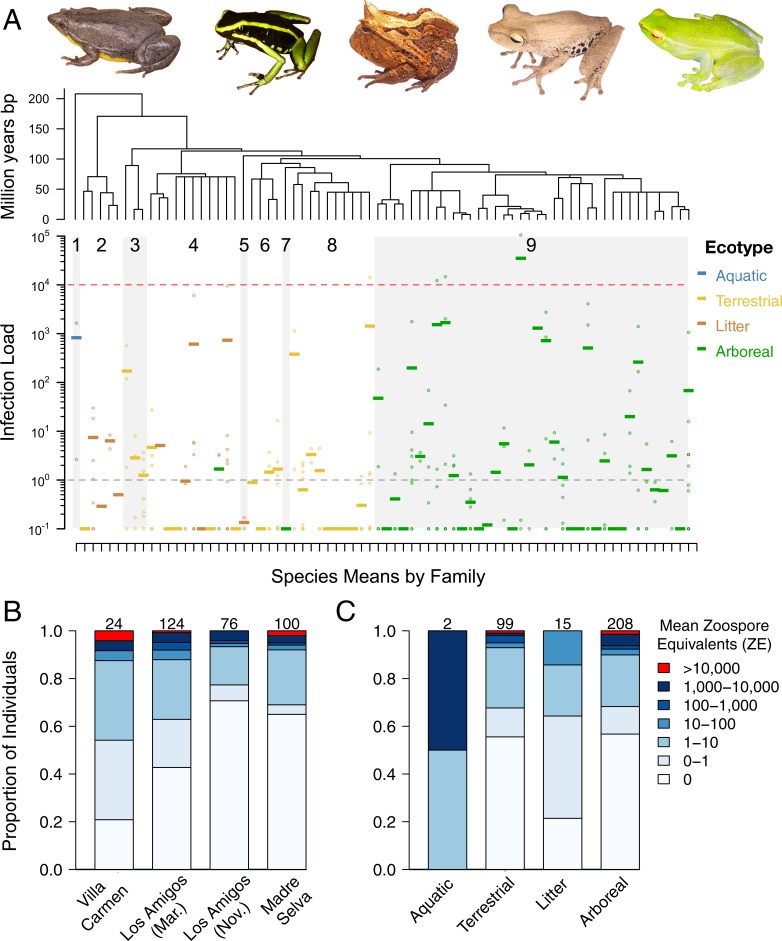
Widespread high infection loads were not predicted by host or site characteristics. (A) Mean individual (points) and mean species (horizontal dashes; *N* = 75 species) log_10_-transformed infection loads (note log scale on the y-axis), ordered by phylogeny (from ref. 36) and colored by ecotype, with representative frog species shown above. Tree branches correspond to each species below. Grey polygons correspond to alternate families, as numbered: 1) Pipidae, 2) Microhylidae, 3) Dendrobatidae 4) Craugastoridae, 5) Ceratophryidae, 6) Bufonidae, 7) Centrolenidae, 8) Leptodactylidae, 9) Hylidae. Horizontal grey dotted line at 10^0^ denotes the load threshold for categorization as a positive infection, horizontal red dotted line at 10^4^ denotes the load threshold for categorization as a heavy infection (ZE > 10,000). Frog images from left to right: *Elachistocleis bicolor* (Microhylidae), *Ameerega trivittata* (Dendrobatidae), *Ceratophrys cornuta* (Ceratophryidae), *Hypsiboas maculateralis* (Hylidae), and *Sphaenorynchus lacteus* (Hylidae), with credit for all images to Daniel L. Rabosky. (B) Proportion of individuals at each infection level by collection site. Numbers at top of each bar denote sample sizes. (C) Proportion of individuals at each infection level by ecotype.

Despite seeing no evidence of frog die-off events or morphological signs of chytrid infections while collecting frogs in the field, we found widespread presence of Bd infection across lowland sites in Peru. Of the total 324 samples tested, 106 were positive for Bd infections (zoospore equivalents [26] (ZE) > 1; Fig 2; S1 Table), giving an overall infection prevalence of 0.34. Raw infection prevalence varied by site and elevation, with 0.46 at Villa Carmen (*N* = 24 total frogs tested), 0.37 at Los Amigos at the end of the wet season (*N* = 124 frogs), 0.24 at Los Amigos at the beginning of the next wet season (*N* = 76 frogs), and 0.31 at Madre Selva (*N* = 100 frogs, S1 Table, Fig 3). Villa Carmen and Madre Selva had higher variance in infection prevalence and smaller sample size than Los Amigos. Mean infection load across the entire dataset (including negative infections) was 566 ZE, with a maximum individual load of over 105,000 ZE. The highest intensities detected were at Villa Carmen, which had infection loads ranging from 0–105,232 ZE (mean = 4447.12 ZE). Los Amigos at the beginning of the wet season had the lowest mean infection intensity (mean = 93.9 ZE), with infection loads ranging from 0–4087.5 ZE. Although Villa Carmen had the highest infection prevalence across sites, this was not significantly higher than other sites (binomial test, *P* = 0.08) and sites did not differ from each other (*χ*^2^ = 12, d.f. = 9, *P* = 0.213).

**Fig 4:**
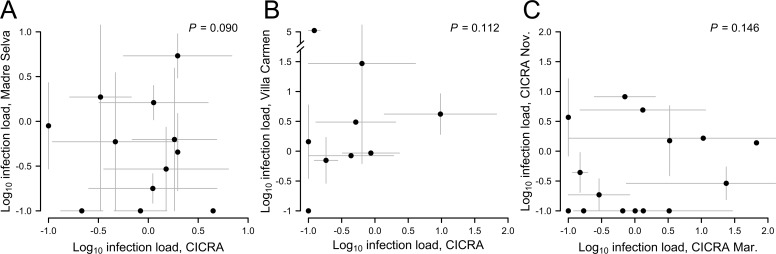
Species-specific infection loads cannot be used to predict infection across sites or seasons. (A) Species mean infection loads (± 1 S.E.M, grey lines) plotted by pairwise site comparisons of the same species (*N* = 12 shared species) captured at Madre Selva and Los Amigos and (B) Villa Carmen and Los Amigos (*N* = 9 shared species) both show no relationship. Note broken y-axis in panel B to allow the display of one very highly infected species at Villa Carmen. Madre Selva and Villa Carmen were not compared because they shared only one species in our dataset. (C) Species mean infection loads plotted by pairwise seasonal comparisons between Los Amigos collections in March 2016 and November 2016 (*N* = 17 shared species) show no relationship. *P*-values are from phylogenetically-informed paired t-tests.

Infection loads reached high levels (critical threshold of ZE ≈ 10,000; see Vredenburg et al [27]) in at least some individuals at all sites (*N* = 4 individuals, Fig 2). However, these loads had no phylogenetic signal (lambda = 0.211, *P* = 1.0; Fig 3A) and we detected positive infections across representatives from nearly all families (80 species total). Infection loads did not vary significantly by site (adj R^2^ = 0.006, *F*_2,311_ = 1.942, *P* = 0.145) but did by season (adj R^2^ = 0.021, *F*_1,193_ = 5.21, *P* = 0.021), at least within our limited sampling design using traditional parametric statistics. Daily precipitation at Los Amigos fluctuated, with mean daily levels decreasing at the end of the wet season and increasing at the beginning of the wet season (January 2016: 9.65 mm; March: 6.8mm; November: 4.51mm; December: 12.62mm; January 2017: 10.21mm; Fig 2A) However, pairwise comparisons of sites and seasons including only shared species assemblages showed no relationship for either site or season (paired t-tests: Los Amigos × Villa Carmen: t = 1.76, *P* = 0.117; Los Amigos × Madre Selva: t = -1.19, *P* = 0.261; Los Amigos both seasons: t = 1.52, *P* = 0.149), even when accounting for phylogeny (phylogenetic paired t-tests: Los Amigos × Villa Carmen: t = 1.86, *P* = 0.112; Los Amigos × Madre Selva: t = -1.90, *P* = 0.09; Los Amigos both seasons: t = 1.54, *P* = 0.146; Fig 4). Generally, we found that the infection load of a species in one site or sampling time point was not correlated with the infection load of the same species at any other site or time point.

Although we found a weak effect of ecotype on infection load using traditional linear models (adj R^2^ = 0.01, *F*_3,310_ = 2.46, *P* = 0.063), this effect did not remain when controlling for phylogenetic effects (phylolm: *t* = 0.138, *P* = 0.89) or accounting for sampling biases (permutation test: z = 1.13, *P* = 0.25). Infection prevalences between terrestrial, burrowing, and arboreal ecotypes were similar (0.32, 0.4, 0.32), while aquatic species had an infection prevalence of 1 (although we had only two aquatic samples representing one species, *Pipa pipa*, Fig 3A, Fig 3B). Results from our ANOVA testing the effect of species on infection loads indicates that only 24% of variance in mean infection loads is explained by species (*P* = 0.4). We found no correlation between SVL and mean infection loads (Spearman’s ρ = 0.009, *P* = 0.88), with SVL accounting for 0.05% of the variance in mean infection loads (adj R^2^ = -0.003, F_1,260_ = 0.13, *P* = 0.72). The phylogenetic generalized least squares showed no independent effect of SVL on infection load (t = -1.4, *P* = 0.16). We found a correlation between sex and infection loads (adj R^2^ = 0.023, F_1,124_ = 3.9, *P* = 0.05), with female prevalence at 0.295 (mean = 2.2 ZE, *N* = 44) and male prevalence at 0.341 (mean = 311.9, *N* = 82). We found frog body and substrate temperatures to be closely paired (*N* = 78; Fig 1B), with most having temperatures cooler than Bd’s CT_max_. Five samples had a >2°C difference between body and substrate temperatures (*Chiasmocleis royi*, *Dendropsophus minutus*, *Rhinella margaritifera*, and *Scinax garbei*), and 7 frogs from one night had body temperatures above CT_max_ of Bd (*Dendropsophus parviceps*, *Dendropsophus sarayacuensis*, *Osteocephalus leprieurii*, *Phyllomedusa camba*, and *Phyllomedusa palliata*).

A total of 28 Bd samples were successfully amplified by PCR using Bd-specific primers and Sanger sequenced for Bd genotype. Based on the comparison of 7 MLST (multilocus sequence typing) loci to reference genomes, all Bd samples sequenced were identified as belonging the global pandemic lineage (GPL). From analysis of hypervariable regions, 3 GPL genotypes are present in the sampled areas. GPL-A was found at all 3 sites in a range of genera (*Pristimantis*, *Ameerega*, *Allobates*, *Scarthyla*, *Hypsiboas*, *Phyllomedusa*, *Dendropsophus*, *Osteocaphalus*, *Edalorhina*, *Leptodactylus*, *Hamptophryne*, *Syncope*, *and Rhinella*). GPL-B was found only in one individual at Villa Carmen (*Osteocephalus castaneicola*), the same individual that had the highest infection load, while GPL-C was found in 1 individual at Los Amigos (*Pristimantis reichlei*).

## Discussion

Although other studies have reported Bd in the Neotropical lowlands (e.g., Costa Rica [28], Panama [29, 30]), infection loads are usually less than a maximum of ~50 ZE, while we found multiple individuals at different sites with critically high infection intensities (ZE > 10,000). Our measured prevalences at all sites during both seasons are also much higher than previously reported for Peru (0.37 in this study vs. 0.01 [23] and 0.045 [24]) or Costa Rica (0.073 [28]), showing a surprising 8-fold increase at Los Amigos alone between 2014 and 2016. Based on this series of results, it is difficult to conclude whether infection prevalence is truly increasing over time or if significant historical infections were undetected. Some of the discrepancy in prevalence may be explained by the different extraction methods used across studies (a PrepMan DNA extraction protocol [23, 24] vs. our higher-yield Qiagen DNeasy protocol), as well as the potential for detection error across studies using skin swabs [31]. However, if this is truly the first documentation of a novel outbreak in Bd prevalence in lowland Amazonia, then our study provides critical and timely information for tropical amphibian conservation biologists and warrants increased surveillance of the lowlands as the outbreak develops. This study serves as the first documentation of Bd genotyping in Peru, despite severe amphibian die-offs due to chytridiomycosis [8, 32]. We detected 3 genotypes of Bd-GPL at our lowland sites, a lineage which has previously been described as generally the most virulent and widespread [22], compared to more endemic lineages. Bd-GPL has been documented on all continents where amphibians occur [33], is hypothesized to have originated in Asia [18], and has been attributed to all chytridiomycosis-caused amphibian die-offs [33]. However, Bd-GPL has not been universally demonstrated to be hypervirulent, as this trait can vary between strains belonging to the same lineage [18, 19]. Because these genotypes exist outside the Bd thermal optimum, they may have unique virulence properties. To determine the virulence of these Peruvian genotypes, isolates should be obtained from these field sites to conduct physiological and transcriptomics experiments.

The high prevalence of Bd infections that we have documented in the western Amazon deserves further exploration, especially in comparison to other lowland sites or time points. Zumbado et al. [34] found high prevalence of low intensity Bd infection across frog species in lowland Costa Rica, with populations showing enzootic disease dynamics, while von May et al. [24] found low prevalence of Bd infections in lowland Peru. They both found that microclimate was the main predictor for Bd infection, with these heterogeneous microclimates offering suitable conditions for Bd within the otherwise unsuitable landscape. While there have been no documented frog declines at our Peruvian collection sites, these populations may play an important role in disease dynamics moving forward. If this pattern is due to either host immunity [12, 14] or heat-induced pathogen weakening [4, 21], these frogs may be either resistant or tolerant of Bd infections [35]. Although resistant individuals may act as a sink for Bd zoospores, tolerant species can harbor infection and facilitate zoospore propagation [36]. Even if they do not show symptoms of disease, they can act as Bd reservoirs or amplification hosts and play key roles in disease outbreaks and persistence. The lack of correlation between infection loads in species that occur at multiple sites is likely due to the variability in host susceptibility between populations of the same species. This wide variability in host susceptibility may be driven by an interaction between host genetics, environment, and local adaptation of Bd strains [19]. Studies highlighting immune-related gene-expression of infected amphibian hosts and local Bd strains would elucidate which processes are responsible for these differences in infection loads across sites.

This study provides critical surveillance data for infection presence and intensity in cold-spots of symptomatic chytridiomycosis, which are severely lacking in the Bd literature. Generally, researchers have primarily tested for Bd in locations where die-off events are observed, but our understanding of this disease relies on the collection of surveillance data across a range of environmental and ecological conditions. We recommend that future studies incorporate swabbing for Bd as a standard part of general museum collection or field ecology studies of amphibians [37]. Through intensive sampling of both Bd hot- and cold-spots around the world, we can learn more about what drives disease dynamics in various systems and apply these insights to other emerging infectious diseases.

## Methods

### Field sampling

We collected skin swab samples from frogs over four collection expeditions, at either the beginning of or during the rainy season (December-May). We collected swabs from three sites in the Peruvian Amazon (Fig 1A). We sampled the foothills site, Villa Carmen Biological Station (up to 850m elevation, latitude: -12.89, longitude: -71.40) from January-February 2016. We sampled one lowland site, Los Amigos Biological Station (270m elevation, 12.56, -70.10) across two collection expeditions in March 2016 and November-December 2016 and the other lowland site, Madre Selva Biological Station (100m elevation, -3.69, -72.46), in January 2017. We also collected thermal data on a) frog body surface temperature and b) temperature of the substrate in physical contact with each frog’s ventral surface using an infrared temperature sensor (Raytek Raynger ST81) during a follow-up trip to Los Amigos in November 2017 (Fig 1B). Daily precipitation from January 2016-February 2017 was collected using All Weather rainfall gauges at Los Amigos (Fig 2A).

We used opportunistic surveys, transects, and standardized survey plots to collect and swab a total of 324 frogs from 80 species. We captured each frog using a plastic bag and used sterile cotton swabs (Dryswab) to collect skin samples[26]. We stored swabs in microcentrifuge tubes containing either 100% ethanol or RNAlater and kept at ambient temperature in the field until transportation to a -20°C freezer at the Museo de Historia Natural Universidad Nacional Mayor de San Marcos (MUSM) in Lima and then until storage at -20°C at the University of Michigan campus. Upon field collection, we recorded the location, species, and sex, mass, and snout-vent length (SVL) of each frog when possible (S1 Table).

### DNA extraction and quantification of infection loads

We extracted DNA using the Qiagen DNeasy Blood and Tissue Kit following the manufacturer’s protocol with modifications for Bd swabs to give a final elution volume of 200uL (dx.doi.org/10.17504/protocols.io[6zhhf36]). Extractions were stored at -20°C. To quantify infection loads, we performed real-time TaqMan PCR (QuantStudio3, Applied Biosystems, dx.doi.org/10.17504/protocols.io.[6zehf3e]) using a fluorescent, Bd-specific probe (Life Technologies; S2 Table), with a total reaction volume of 25uL per well and reported infection loads as ZE (zoospore equivalents) [26]. Each sample was run in triplicate, and samples were classified as positive for infection if they had a mean ZE > 1. Each plate included both a negative control and a set of positive standards (either CLFT 035 or CLFT 073, Brazilian Atlantic Forest strains) diluted to give 10^6^−1 ZE concentrations, from which a standard curve was generated. We calculated infection prevalence as the number of individuals with ZE > 1 divided by the total number of individuals tested for infection, either overall or by study site.

### Identification of pathogen strain

To identify fungal genotype of Bd positive samples, we used multilocus sequence typing (MLST, dx.doi.org/10.17504/protocols.io.[6zghf3w]) to target lineage-informative regions of the Bd genome. We first amplified DNA using ExTaq Polymerase (TaKaRa) and previously described lineage-informative primers: 8009X2 [38]; BdC5 [39]; BdSC3.1, BdSC4.16, BdSC6.15 [40]; BdSC6.8 [41], and R6064 [42] (S2 Table). To amplify these loci except BdSC6.8, PCR conditions were as follows: 1 cycle of 94°C/3 min; 45 cycles of 94°C/1 min, 54°C/30 sec, 72°C/1 min; 1 cycle of 72°C/7 min. To amplify BdSC6.8, the previous PCR conditions were the same except for an increased annealing temperature of 60°C [41]. Amplified PCR products were purified using a 1/11 dilution of ExoSAP-IT (Affymetrix) and Sanger sequenced on both strands (3730xl DNA Analyzer, Applied Biosystems) at the University of Michigan DNA Sequencing Core. To determine Bd strain and lineage, we aligned sequences in each direction for each sample and compared these contigs to reference genotypes [41] in Sequencher v 4.10.1 (GeneCodes).

### Molecular identification of unidentified frogs

To identify a subset of frogs that could not be confidently identified during collection (*N* = 6 samples), we amplified the 16S region of the genome from the swabs using primers 16SAR and 16SBR (S2 Table) [43] with PCR conditions as follows: 1 cycle of 96°C/3 min; 35 cycles of 95°C/30 sec, 55°C/45 sec, 72°C/1.5 min; 1 cycle 72°C/7 min. Amplified products were purified and sequenced using the same methods as for the MLST Bd markers. To determine frog species, we aligned sequences in each direction for each sample and compared contigs to reference sequences available in GenBank and some obtained more recently (R. von May et al, unpublished; S4 Table) using Geneious R6 v 6.1.8 (Biomatters 2013).

### Statistical analyses

We tested for three categories of effects on infection load: differences among sites, differences across seasons at Los Amigos, and differences associated with host phenotype (host ecotype, species, SVL, and sex). For these analyses, the dataset was pruned to include only species found in the tree generated by Jetz and Pyron [44] (*N* = 75 species and 314 individuals). Host ecotypes were assigned by frog genus following IUCN habitat and ecology descriptions supplemented with additional primary sources (see S3 Table). The “litter” ecotype denotes ground-dwelling species that primarily live amongst leaf litter. For all analyses of infection load, we added a small constant (0.1) to each mean infection load before logarithmic (log_10_) transformation to allow statistical comparison of all samples. All tests were run in R v3.4.3 and significance was assessed at *P* < 0.05. First, we assessed phylogenetic signal using a lambda test implemented in the ‘phytools’ package [45]. For site and season, we ran standard linear models and both standard and phylogenetic paired t-tests in ‘phytools’.

We also tested the difference in infection prevalence between sites. Because we chose non-phylogenetic tests for prevalence, we used the full dataset of 80 species. We treated the two seasons of Los Amigos as two independent samples because of the marked differences in temperature and rainfall. We first ran a Chi^2^ test of independence to test the difference in infection prevalences between sites. We also ran a binomial test to calculate the probability of obtaining the observed between-site prevalence by chance if we had sampled each site as little as we did Villa Carmen.

To test for the effect of host ecotype, we used both standard linear models and phylogenetic linear models in the ‘phylolm’ package [46]. We also ran a permutation test to test the magnitude of infection load differences between ecotypes and one-way ANOVA to determine the percent variance in infection loads between host species. For SVL effects, we conducted standard linear models, nonparametric Spearman’s rank test, and a PGLS. Lastly, we ran a linear model to test for the effect of host sex on infection load.

### Ethics statement

All methods were approved by the University of Michigan’s IACUC (Institutional Animal Care and Use Committee) under protocol #PRO00006234. Specimens were collected and exported according to the SERFOR (Servicio Nacional Forestal y de Fauna Silvestre) permitting requirements (permit numbers: 029-2016-SERFOR-DGGSPFFS, 405-2016-SERFOR-DGGSPFFS, 116-2017-SERFOR-DGGSPFFS) and imported according to the United States Fish and Wildlife Service permitting requirements.

## Supporting information

S1 Table Specimen data. Voucher numbers, ecotype, morphological data, and infection loads for Peruvian frog specimens used in analysis.
(XLSX)

S2 Table Primer sequences. Primers used in PCR and sequencing.
(XLSX)

S3 Table Ecotype assignments and references. References for species assignment to ecotype classifications of terrestrial, litter, arboreal, and aquatic.
(XLSX)

S4 Table GenBank accession numbers. GenBank numbers for comparison sequences used to assign 6 unidentified frogs specimens to species.
(XLSX)
